# Analyzing to discover origins of CNNs and ViT architectures in medical images

**DOI:** 10.1038/s41598-024-58382-3

**Published:** 2024-04-16

**Authors:** Seungmin Oh, Namkug Kim, Jongbin Ryu

**Affiliations:** 1 Department of Artificial Intelligence, Ajou University, Suwon, South Korea; 2 Department of Convergence Medicine, Asan Medical Center, University of Ulsan College of Medicine, Seoul, South Korea; 3 Department of Software and Computer Engineering, Ajou University, Suwon, South Korea

**Keywords:** Biomedical engineering, Electrical and electronic engineering

## Abstract

In this paper, we introduce in-depth the analysis of CNNs and ViT architectures in medical images, with the goal of providing insights into subsequent research direction. In particular, the origins of deep neural networks should be explainable for medical images, but there has been a paucity of studies on such explainability in the aspect of deep neural network architectures. Therefore, we investigate the origin of model performance, which is the clue to explaining deep neural networks, focusing on the two most relevant architectures, such as CNNs and ViT. We give four analyses, including (1) robustness in a noisy environment, (2) consistency in translation invariance property, (3) visual recognition with obstructed images, and (4) acquired features from shape or texture so that we compare origins of CNNs and ViT that cause the differences of visual recognition performance. Furthermore, the discrepancies between medical and generic images are explored regarding such analyses. We discover that medical images, unlike generic ones, exhibit class-sensitive. Finally, we propose a straightforward ensemble method based on our analyses, demonstrating that our findings can help build follow-up studies. Our analysis code will be publicly available.

## Introduction

In medical image recognition, analyzing the decision-making process of deep learning is very critical. The reliability of deep learning will drop if it can’t be analyzed in the decision-making process in determining a disease. Li et al.^1^ explore the potential of utilizing a Vision Transformer (ViT) in medical data and compare its performance to that of Convolutional Neural Networks (CNNs). A recent study discovered that artifacts present in medical datasets can greatly affect the accuracy of classification models^2^. In addition, a study by Raghu et al.^3^ examined the factors to be considered when applying the transfer learning method from general images to medical images.

Despite this, while quite a few studies of deep learning analysis have been conducted on generic images, such as ImageNet^4^ dataset, it has not yet been extensively investigated in medical image recognition. Several approaches^5–10^ for examining deep neural networks have been done on the generic dataset; such as texture-shape analysis, robustness, translation invariant consistency, and frequency analysis, they are still insufficient in the medical data for these analyses. To this end, in this paper, we introduce novel medical data analysis through extensive and well-designed experiments. Specifically, we aim to investigate the grounds of performance difference between the convolutional neural networks (CNNs) and vision transformer (ViT) with our analysis.

The research on whether to employ CNNs or ViT is still a contentious topic in both generic and medical images. Only a few studies^1,11,12^, however, have been done in the medical image domain. We analyze robustness, translation-invariance, obstruction, and shape-texture bias by redesigning the analyses done on generic images to fit medical images. This paper will identify the origins of CNNs and ViT in order to provide intuition for future research. We investigate robustness^7,9^, translation invariance^10^, obstruction, and shape-texture bias^7,8^ in medical images by revamping analyses performed on generic images. The contribution of our paper is summarized as follows:

- We, in this paper, reveal the origin of CNNs and ViT model performance in medical images. To provide insightful analysis, we conduct extensive experiments that influence the recognition performance of medical images, such as shape and texture bias.
- We demonstrate a notable difference in performance by class labels in medical images. This finding illustrates the specific property that medical image is especially class-sensitive.
- We propose a new classification method dubbed class-conditional ensemble based on our findings. Using a simple strategy, the proposed ensemble method improves the performance of all metrics.

## Background

**CNNs** have long been the most outstanding visual recognition architecture  ^13–17^. Extracting regional features with a convolutional kernel learns the strong correlation between surrounding pixels, and thus, inductive biases such as translation invariance and equivariance can be learned effectively. However, due to the limited kernel size, the convolution operation suffers from learning the global features that lead to reaching the limit of performance improvement. To overcome this limitation,  ^18–21^ use $1 \times 1$ convolution to reduce focusing only on local information or do re-calibration through channels operation.

**Vision Transformer** ^22–25^ uses self-attention to learn the association between all pixels globally, unlike convolution, which only considers surrounding pixels. This is an entirely different learning process than existing CNN-based models, so many studies have begun to employ self-attention. Although the performance of ViT cannot be stated to be high in an insufficient quantity of training data, lots of effort is still being undertaken ^23,24^ since ViT has a better model capacity in terms of learning global information.

**Analytic study on deep neural networks** ^5–7^ to the generic images (*i*.*e*., ImageNet ^4^) has been conducted to analyze CNNs and ViT. When adequate training data is available, ViT has a lower risk of falling into a local minimum. However, when the training data is insufficient, CNNs can readily learn the inductive bias well and perform better than ViT. To complement these two architectures, studies on hybrid models are also increasing. Several studies ^1,11,12^ in the medical domain examine the performance of CNNs and ViT, as well as the effect of transfer learning with ImageNet pre-trained architecture. Raghu et al. ^3^ raised concerns about over-parameterization when applying transfer learning to medical datasets, given the small amount of training data. In a recent study, Juodelyte et al. ^26^ put forward a method to enhance the resilience of transfer learning in medical data, specifically addressing the challenge of out-of-distribution data. Examining the issue of medical dataset composition, a study by Bissoto et al. ^2^ found that artifacts present in the skin image datasets ^27,28^ have a notable influence on visual recognition. The research conducted by Sun et al. ^29^ explored the impact of training with corrupted images and the extent to which models relied on these artifacts.

## Settings

We conduct various experiments using CNNs, ViT, and hybrid architectures to explore their respective characteristics. Additionally, we investigate how their strengths are leveraged across multiple forms of data. All training is done using the AdamW optimizer with a learning rate of 0.0001 and cosine annealing scheduler. The loss function is binary cross entropy, and the input image size is $224 \times 224$. All the experiments were conducted using 5-fold cross-validation. The numbers in brackets in the experimental results represent the 95% confidence interval for the results of the five validations. In addition, unless specified otherwise, we use AUROC to evaluate the classification performance.

### Architecture

The experiment is divided into CNNs, ViT, and hybrid, because this analysis is to find the origin of CNNs and ViT architectures in the medical data. We select CNN-based ResNet^13^ and DenseNet^15^, which are commonly used in medical data. For the ViT architecture, DeiT^24^ and Swin-Transformer^23^ are utilized, while CoAtNet^25^ and MaxViT^30^ are chosen as hybrid architecture. We attempt to select backbones with similar parameters to ensure a fair evaluation and backbones are trained as classification tasks.

### Dataset

The dataset is used differently depending on the analysis. CheXpert^31^ is used in robustness and consistency analysis, ChestX-ray14^32^ is used for obstruction analysis, and ISIC2017^33^ is used to find texture and shape bias.

**CheXpert**^31^ is a large chest radiograph dataset. It consists of 224,316 chest radiographs for training and 200 validation radiographs. And the labels of train data are made into natural language processing and the labels of validation data are made by experts. In our analysis, we train the compare groups as a multi-label task using five pathology classes: Cardiomegaly(Cd.), Edema(Ed.), Consolidation(Co.), Atelectasis(A.), and Pleural Effusion(P.E.).

**ChestX-ray14** is an extension of ChestX-ray8^32^ by adding six additional thorax lesions. It is a chest X-ray dataset comprised of frontal-view radiograph images with fourteen lesion labels. The labels are made into natural language processing from the associated radiological reports. The datasets for training and validation have sizes 86524 and 25596, respectively. In our analysis, we train the compare groups as a multi-label task using 14 classes. Also, we use bounding box annotation data that consists of 8 classes for performance measuring.

**ISIC2017**^33^ is a skin lesion dataset comprising 2000 training images and 150 validation images with 3 classes and segmentation annotation of lesion area. In our analysis, we train the compare groups as a multi-class task using three classes.

## Empirical study

In order to ensure fair comparisons, we use similar scales of networks of CNNs and ViT. Table 1 provides the number of parameters utilized in our experiments.

**Table 1:** Performance and size of models on CheXpert. The terms ’Clean’ and ’Corrupted’ refer to the quality of the original data, indicating whether it is free from any corruption or if it has been corrupted, as mentioned in Section 'Robustness'. Under the double line is the average of the models representing each architecture.

Architecture	# of Params	Clean	Corrupted
ResNet50	22.43	0.87 (0.00)	0.78 (0.01)
DenseNet201	17.26	0.87 (0.00)	0.81 (0.01)
DeiT Small	20.66	0.88 (0.00)	0.81 (0.00)
Swin Tiny	26.25	0.88 (0.00)	0.81 (0.00)
CoAtNet 0	25.44	0.87 (0.00)	0.80 (0.01)
MaxViT Tiny	27.22	0.88 (0.00)	0.81 (0.01)
CNNs	19.845	0.872	0.794
ViT	23.455	0.878	0.811
Hybrid	26.330	0.875	0.804

### Robustness

**Figure 1:**
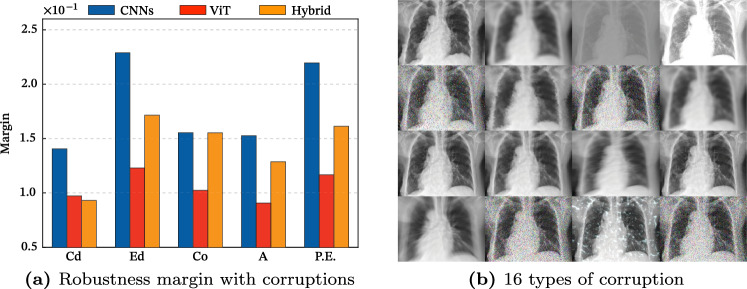
Experimental results and examples of the robustness with 16 types of corruption. A big margin indicates a considerable deterioration in performance due to corruption. We take this finding to mean that ViT is more resistant to corruption than CNNs. It is also worth noting that Edema and Pleural Effusion are more susceptible to corruption than other lesions.

The robustness analysis^7,9^ investigates the performance deterioration when various corruptions are applied to the medical images. A total of 16 forms of corruption(Fig. 1b) are employed, including brightness, elastic transform, lossy compression, and Gaussian blur, with corruption intensities ranging from I1 to I5. I1 and I2 are the levels of corruption that typically exist in the real world. Extreme corruption, l3 to l5, can also occur in images of very fat or thin people as a limit to the visualizable area of the image histogram. It is evident that a model’s ability to handle corrupted data directly impacts its performance with real-world medical images. Due to certain patients’ inability to hold their breath or maintain stillness during X-ray procedures, the resulting images may become distorted. In addition, variations in noise and brightness can occur due to differences in the patient’s body form, as the medical equipment is not calibrated individually for each patient. Given the potential for medical images to be corrupted in various real-world settings, this paper conducts a robustness analysis. We exhibit the performance gap of AUROC based on the intensity of architectures. This gap between clean (*i*.*e*., original data) and corrupted data can be measured to validate the robustness. The absolute performance of CLEAN is better for CNNs, which is consistent with previous research^34^ showing that CNNs generally perform better in noiseless settings. Therefore, rather than comparing absolute performance, we study which architecture is more noise-robust by measuring the amount of performance degradation. As the level of corruption grows in all backbones, so does the performance degrades in Table 2. CNNs are the least resilient to corruption data, while ViT is the most robust under the same corruption data. On the other hand, on the clean dataset, CNNs outperform the ViT architecture considerably due to their efficient convolution operation. Another intriguing fact is that performance differences between classes within a single dataset can be significant. The Fig. 1a shows the margin by class. It can be seen that Edema and Pleural Effusion have a significant performance gap when compared to other classes. This is interpreted as follows for two reasons. The first is that Edema and Pleural Effusion have low texture information compared to other classes in the dataset. The second reason is that CNNs learn more texture information than ViT. As a result, texture corruption considerably reduces the performance of the texture-sensitive Edema and Pleural Effusion classes for CNNs. The correlation between CNNs and textures is more detailed in Section 'Shape and texture bias'.

**Table 2:** Performance degradation of the corruptions with five intensities on CheXpert. The Archi. denotes architecture. We conducted the experiment using a 5-fold cross-validation method. The numbers in parentheses represent the 95% confidence interval.

Archi.	$\Delta$AUROC$\downarrow$($\text {AUROC}_{clean}-\text {AUROC}_{corrupted}$)					
	I1	I2	I3	I4	I5	Mean
Res.	0.03 (0.00)	0.05 (0.01)	0.07 (0.01)	0.12 (0.02)	0.17 (0.02)	0.09 (0.01)
Dense.	0.01 (0.00)	0.03 (0.00)	0.05 (0.00)	0.10 (0.00)	0.14 (0.01)	0.07 (0.00)
DeiT	0.02 (0.00)	0.03 (0.00)	0.05 (0.00)	0.09 (0.01)	0.13 (0.01)	0.06 (0.00)
Swin	0.02 (0.00)	0.04 (0.00)	0.06 (0.00)	0.09 (0.01)	0.14 (0.01)	0.07 (0.01)
CoAt.	0.02 (0.01)	0.04 (0.01)	0.06 (0.01)	0.11 (0.01)	0.16 (0.01)	0.08 (0.01)
Max.	0.01 (0.01)	0.03 (0.01)	0.04 (0.01)	0.09 (0.02)	0.15 (0.01)	0.06 (0.01)
CNNs	0.021	0.039	0.060	0.110	0.157	0.077
ViT	0.019	0.037	0.057	0.090	0.133	0.067
Hybrid	0.015	0.034	0.053	0.100	0.157	0.072

### Consistency of translation invariance

**Figure 2:**
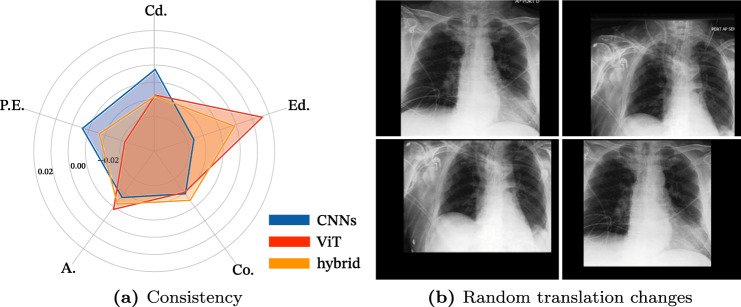
Experimental result and examples of consistency analysis on the translation invariant property. a) It shows the per-class performance deviation of the consistency. Interestingly, each design of CNNs and ViT outperforms the others in different classes, demonstrating that architectural choice is critical in medical image recognition. b) The left-top is an original image, and the others are randomly translated from the original image.

Translation invariance is an important property that allows the model to recognize an object regardless of its translation changes^10^. Two random translation changes are randomly added to the original images for evaluating the translation invariance property, as seen in Fig. 2b. We measure the model’s translation invariance ability using the recognition consistency of these two translation changes, and its formula is defined as $Consistency = \frac{1}{N}\sum _{i} \mathbbm {1} \, \{y^1_{i}=y^2_{i}\},$ where $y^1_{i}$ and $y^2_{i}$ are labels of randomly translated two images from original images that have $y_i$ labels. *N* denotes the number of images, and *y* represent the label of images. We ensure that no more than 7% of the image is lost during this translation change to preserve the lesion information. There are distinctive cases in medical images where a lesion has a fixed onset location or appears randomly in multiple locations. As a result, the significance of translation invariance changes based on the types of diseases, and if the appropriate analysis is applied, the performance can be enhanced further. In Table 3, the consistency of the translation invariant property is high in the order of CNNs, hybrid, and ViT. The highest consistency of CNNs is due to the pooling layer of its architectural design and is consistent with earlier studies^35^. On the other hand, unlike the CNNs having a pooling layer, ViT consists of only the self-attention layer that encodes global pixel interactions without the pooling layer. However, as demonstrated in Fig. 2a, in three classes, Cardiomegaly, Edema, and Atelectasis, the translation invariance property is less important because the site of one is constant for these three lesions. As a result, in these classes, ViT outperforms CNNs architecture.

**Table 3:** Consistency performance against the translation invariant property on CheXpert. CNNs have been shown to outperform alternative architectures in terms of consistency performance. This finding suggests that the pooling operation of CNNs is highly effective in capturing the translation changes of medical images.

Archi.	Cd.	Ed.	Co.	A.	P. E.	Mean
Res.	0.87 (0.03)	0.90 (0.03)	0.92 (0.02)	0.89 (0.02)	0.87 (0.02)	0.89 (0.02)
Dense.	0.88 (0.02)	0.91 (0.04)	0.88 (0.02)	0.90 (0.03)	0.88 (0.03)	0.89 (0.02)
DeiT	0.84 (0.02)	0.85 (0.04)	0.85 (0.01)	0.84 (0.01)	0.83 (0.03)	0.84 (0.02)
Swin	0.88 (0.03)	0.93 (0.03)	0.90 (0.04)	0.89 (0.02)	0.88 (0.04)	0.90 (0.03)
CoAt.	0.90 (0.03)	0.91 (0.04)	0.91 (0.03)	0.91 (0.04)	0.91 (0.04)	0.91 (0.03)
Max.	0.88 (0.02)	0.90 (0.02)	0.87 (0.03)	0.88 (0.02)	0.88 (0.02)	0.88 (0.01)
CNNs	0.878	0.904	0.898	0.895	0.873	0.889
ViT	0.858	0.890	0.873	0.868	0.855	0.869
Hybrid	0.891	0.905	0.890	0.896	0.896	0.895

**Table 4:** Performance comparison between general and our class-conditional ensemble methods. Our ensemble method performs better than the baseline. The Acc., F1., Spe., Sen., and Pre. represent accuracy, F1 score, specificity, sensitivity, and precision, respectively. In this table, we report the absolute value of each metric.

Method	Acc.	AUROC	AUPRC	F1.	Spe.	Sen.	Pre.
General ensemble	0.743	0.668	0.508	0.320	0.914	0.280	0.502
Class-conditional ensemble	0.744	0.670	0.510	0.329	0.909	0.293	0.503

This finding holds significant importance in developing a suitable method for medical image recognition. We propose a straightforward yet powerful approach to develop a class-conditional ensemble method using CNNs and ViT architecture. Our ensemble method takes a different approach compared to the general ensemble method. Instead of simply summing up the estimated probabilities of its member networks, our method uses the deviation of consistency to incorporate the estimated probability of each architecture conditionally. Specifically, we give more weight to the estimated probability of the three translation-change insensitive classes to the ViT architecture. We assigned a weight of 0.75 to the translation-change insensitive classes and a weight of 0.25 to the rest classes for the ViT architecture. This weighting value is applied in reverse to CNNs. Note that this is only to demonstrate the possibilities that our analysis can provide for future studies; we do not utilize a sophisticated learning algorithm. Nevertheless, our class-conditional approach performs better than the general ensemble method, as shown in Table 4.

### Obstruction

**Figure 3:**
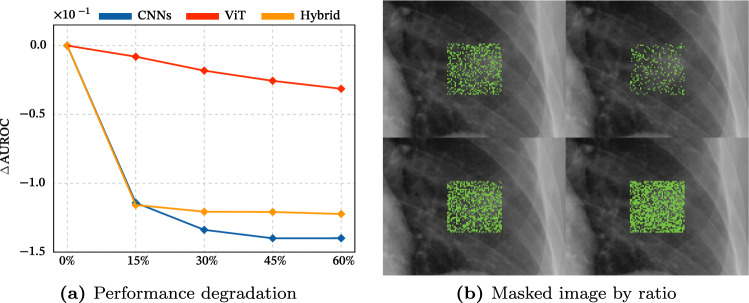
Experimental results and examples of the obstruction analysis. (**a**) As the masking ratio of the obstruction increases, the performance degrades while the degradation of ViT is much smaller than others. (**b**) The left-top is the cases where the masking ratios are 15%, 30%, 45%, and 60% in clockwise order on the ChestX-ray14 dataset.

Efforts to apply deep learning in medical images have long been made, but there are numerous issues that must be resolved before they can be employed in the real world. For example, there is a significant discrepancy between the image used for training deep neural networks and images utilized for diagnosis in the real world. Publicly accessible datasets often contain images of diseases that are easily recognizable. However, real-world images are captured under various conditions, which may result in minimal evidence of the disease. In this section, we will be examining how the model’s performance is affected when certain regions of the lesion are intentionally occluded from the image, leaving only a small portion of the lesion visible. To this end, we evaluate architectures on the ChestX-ray14 dataset while occluding random lesion regions of the image, as shown in Fig. 3b. For this experiment, we used bounding box labels provided by the official website (https://nihcc.app.box.com/v/ChestXray-NIHCC). Figure 3a indicates the performance degradation according to the ratio of obstruction regions for each architecture. The most noticeable finding is that ViT has a low-performance degradation rate compared to CNNs and hybrids, which is also consistent with Section 'Robustness' results. The performance degradation in CNNs accelerates as the masking ratio increases. This result is interpreted as being particularly vulnerable to obstruction due to the convolution operation that encodes the association between surrounding pixels. As a result, it is worthwhile to employ ViT in medical images where only a portion of the lesion is visible or when the image is obtained from a different view.

### Shape and texture bias

**Figure 4:**
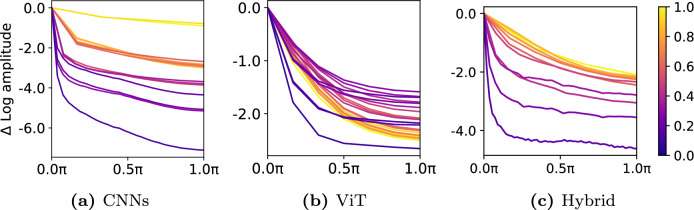
Experimental result of frequency analysis. Each graph depicts the amplitude variation by frequency in the CheXpert dataset. CNNs learn higher frequency features, whereas ViT learns lower ones relatively. In other words, CNNs use texture information to make predictions rather than shape information, but ViT actively utilizes shape information.

**Figure 5:**
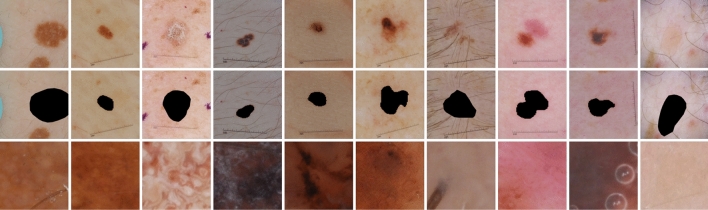
Examples of synthesized images for shape and texture analysis on the ISIC2017 dataset. We remove the color values from each lesion location of the original images (first row), leaving only the shape information of the lesion boundary (second row). To leave the texture information only, we crop the lesion area of the original images and stretch it (third row).

Previous studies^7,8^ have found that shape information, rather than texture, is critical for humans to recognize images. However, deep learning models, particularly CNNs, distinguish images based on texture rather than shape. Since the origin of humans and deep neural networks may differ in understanding images, and even between deep neural networks, depending on the constitution of the architecture, such as CNNs and ViT, as shown in Fig. 4. This difference cannot be conclusively determined in which information should be considered more, however, it can be useful to give a foundation for creating an appropriate algorithm. As a result, regarding the analysis of shape and texture bias, we have conducted validation tests on CNNs and ViT using medical images. We measured the recognition performance specifically on shape-only and texture-only images, as demonstrated in Fig. 5. To eliminate texture information, we synthesized the lesion area. Also, we evaluate the performance of networks using texture-only images. In this experiment, we report performance degradation when using the shape-only images compared to the original clean images. It is noticeable that ViT shows a substantially lower performance degradation than CNNs and hybrid architectures, as shown in Table 5. This means that ViT learns more shape information because the boundary edge of the lesion region still keeps shape information even when the lesion region is veiled. As a result of ViT’s shape-aware property, its performance is better than other architectures. On the other hand, Table 6 shows that CNNs perform better than ViT using only texture images. This result indicates that CNNs are able to learn texture information more effectively. To summarize, ViT performs better on datasets with prominent shape features, while CNNs perform better on datasets with more texture information.

**Table 5:** Experimental results of the shape bias analysis. We present the decrease in performance as $\Delta$AUROC$\downarrow$ ($\text {AUROC}_{clean}-\text {AUROC}_{shape}$) resulting from the use of shape-only images instead of the original ones. We report the average value from the last three rows.

Backbone	Melanoma	Seborrheic Keratosis	Nev	Mean
Res.	0.12 (0.01)	0.36 (0.01)	0.18 (0.01)	0.21 (0.01)
Dense.	0.17 (0.02)	0.23 (0.01)	0.13 (0.01)	0.18 (0.01)
DeiT	0.20 (0.01)	0.20 (0.01)	0.16 (0.01)	0.18 (0.00)
Swin	0.19 (0.02)	0.23 (0.01)	0.14 (0.01)	0.19 (0.01)
CoAt.	0.17 (0.02)	0.25 (0.01)	0.15 (0.01)	0.19 (0.01)
Max.	0.21 (0.01)	0.28 (0.01)	0.13 (0.01)	0.20 (0.01)
CNNs	0.143	0.294	0.156	0.196
ViT	0.198	0.211	0.150	0.186
Hybrid	0.188	0.266	0.138	0.196

**Table 6:** Experimental results of the texture bias analysis. We show the performance decrease as $\Delta$AUROC$\downarrow$($\text {AUROC}_{clean}-\text {AUROC}_{texture}$) resulting from the use of texture-only images instead of the original ones. We present the average value in the last three rows.

Backbone	Melanoma	Seborrheic Keratosis	Nev	Mean
Res.	0.55 (0.03)	0.15 (0.03)	0.36 (0.03)	0.33 (0.02)
Dense.	0.69 (0.06)	0.19 (0.04)	0.49 (0.03)	0.42 (0.04)
DeiT	0.70 (0.03)	0.14 (0.03)	0.49 (0.03)	0.40 (0.02)
Swin.	0.69 (0.04)	0.10 (0.03)	0.64 (0.03)	0.41 (0.02)
CoAt.	1.11 (0.04)	0.31 (0.07)	0.60 (0.04)	0.60 (0.05)
Max.	0.86 (0.03)	0.26 (0.03)	0.54 (0.03)	0.50 (0.02)
CNNs	0.620	0.171	0.425	0.371
ViT	0.696	0.121	0.563	0.404
Hybrid	0.985	0.285	0.570	0.551

## Discussion and conclusion

In this paper, we investigate the origins of medical image recognition in modern deep architectures such as CNNs, ViT, and hybrid. We find their origins using a variety of analyses, including disease classification, robustness, translation invariance, obstruction, and shape-texture bias.

**Figure 6:**
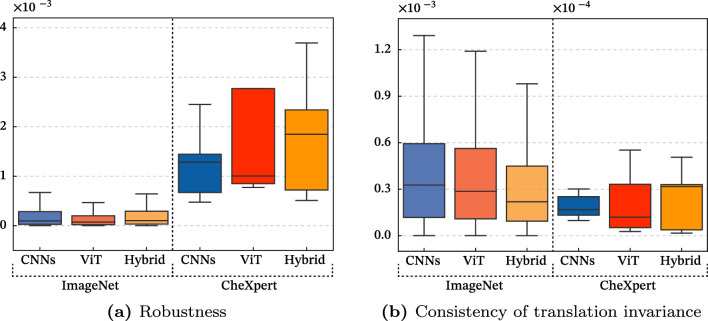
Experimental comparison between generic (*i*.*e*., ImageNet) and medical (*i*.*e*., CheXpert) images. We compare the variance regarding the robustness and consistency of translation invariance for three architectures by class. Medical image data has a higher variance in robustness, while it has a significantly different variance for each class in the consistency value.

In clean images, CNNs outperform ViT when models with similar parameters are used. CNNs have the advantage of being highly resilient to the translation invariant property due to their powerful local convolution operation. Additionally, the global self-attention operation in ViT enhances its robustness. More specifically, as shown in Fig. 6 regarding the robustness and consistency of the translation invariant property, it is worth noting that all architectures show similar variance in generic images (*i*.*e*.ImageNet), but with medical ones, the variances are considerably different depending on the architecture. This is because the unique feature of each lesion class considerably differs in the medical images from the generic images. We summarize our analyses in Table 7; CNNs perform well on clean images due to their strong local operator and have the advantage of translation invariance. ViT, on the other hand, achieves better results on robustness and obstruction analysis, as well as a higher shape bias akin to humans. Despite the worse performance of ViT compared to CNNs with the clean setting, due to insufficient medical images for training a model sufficiently, it is helpful for the real-world scenario under severe noise and wild settings.

**Table 7:** Summary of our finding regarding the origin of CNNs and ViT.

	Clean image	Noise	Translation	Obstruction	Shape-texture
CNNs	accurate $\uparrow$	fragile $\downarrow$	invariance $\uparrow$	fragile $\downarrow$	texture
ViT	in-accurate $\downarrow$	robust $\uparrow$	less-invariance $\downarrow$	robust $\uparrow$	shape

## Data Availability

In this paper, we have used all the publicly available datasets, such as CheXpert, ChestX-ray14, and ISIC2017. Each dataset can be downloaded from their public repository (**CheXpert.** https://stanfordmlgroup.github.io/competitions/chexpert/; **ChestX-ray14.** https://nihcc.app.box.com/v/ChestXray-NIHCC; **ISIC2017.** https://challenge.isic-archive.com/data/).
